# Leaf vein density correlates with crassulacean acid metabolism, but not hydraulic capacitance, in the genus *Clusia*

**DOI:** 10.1093/aob/mcad035

**Published:** 2023-02-23

**Authors:** Alistair Leverett, Kate Ferguson, Klaus Winter, Anne M Borland

**Affiliations:** School of Natural and Environmental Sciences, Newcastle University, Newcastle Upon Tyne, NE1 7RU, UK; Smithsonian Tropical Research Institute, PO Box 0843-03092, Balboa, Ancón, Republic of Panama; School of Life Sciences, University of Essex, Colchester Campus, Colchester, CO4 3SQ, UK; School of Natural and Environmental Sciences, Newcastle University, Newcastle Upon Tyne, NE1 7RU, UK; Smithsonian Tropical Research Institute, PO Box 0843-03092, Balboa, Ancón, Republic of Panama; School of Natural and Environmental Sciences, Newcastle University, Newcastle Upon Tyne, NE1 7RU, UK

**Keywords:** Crassulacean acid metabolism, hydraulic capacitance, xylem, vein density, *Clusia*, leaf anatomy

## Abstract

**Background and Aims:**

Many succulent species are characterized by the presence of Crassulacean acid metabolism (CAM) and/or elevated bulk hydraulic capacitance (*C*_FT_). Both CAM and elevated *C*_FT_ substantially reduce the rate at which water moves through transpiring leaves. However, little is known about how these physiological adaptations are coordinated with leaf vascular architecture.

**Methods:**

The genus *Clusia* contains species spanning the entire C_3_–CAM continuum, and also is known to have >5-fold interspecific variation in *C*_FT_. We used this highly diverse genus to explore how interspecific variation in leaf vein density is coordinated with CAM and *C*_FT_.

**Key Results:**

We found that constitutive CAM phenotypes were associated with lower vein length per leaf area (VLA) and vein termini density (VTD), compared to C_3_ or facultative CAM species. However, when vein densities were standardized by leaf thickness, this value was higher in CAM than C_3_ species, which is probably an adaptation to overcome apoplastic hydraulic resistance in deep chlorenchyma tissue. In contrast, *C*_FT_ did not correlate with any xylem anatomical trait measured, suggesting CAM has a greater impact on leaf transpiration rates than *C*_FT_.

**Conclusions:**

Our findings strongly suggest that CAM photosynthesis is coordinated with leaf vein densities. The link between CAM and vascular anatomy will be important to consider when attempting to bioengineer CAM into C_3_ crops.

## INTRODUCTION

The field of leaf hydraulics has grown in the last 15 years, following the discovery that leaves act as a hydraulic bottleneck in trees, accounting for over 30 % of the hydraulic resistance in the soil–plant–atmosphere continuum (Sack *et al.*, 2003; Sack and Tyree, 2005; Sack and Holbrook, 2006; Scoffoni *et al.*, 2011; Wolfe *et al.*, 2022). More than 40 trillion tonnes of water traverse leaves each year (Chahine, 1992). Before entering leaf mesophyll tissue, water first travels within hollow, lignified tube-like structures, called xylem conduits. Xylem tissue plays an integral role in allowing water to permeate further across the width of a leaf, thereby keeping photosynthetic tissue hydrated. Due to the importance of xylem, and with the growing threat of global warming, it is essential that we understand the relationships between physiological traits and vascular architecture in leaves (Sack and Tyree, 2005; Sheffield and Wood, 2008; Choat *et al.*, 2018; Jiao *et al.*, 2021).

One physiological adaptation that is thought to have a substantial effect on transpiration rates of plants is Crassulacean acid metabolism (CAM). CAM is a photosynthetic mode that minimizes water loss via reorganized stomatal dynamics, so that stomata open at night and close during the day (Borland *et al*., 2014, 2015; Yang *et al.*, 2015; Hurtado-Castano *et al.*, 2023). By keeping stomata closed during the middle of the day (when the air is hottest and driest), CAM plants can decrease transpiration and conserve water. Consequently, CAM prevents leaf water potentials (Ψ_L_) from falling low enough to cause mechanical damage to vascular and mesophyll tissues (Haag-Kerwer *et al.*, 1996; Winter *et al.*, 2005; Leverett *et al.*, 2023). However, despite the substantial effect CAM has on the movement of water through leaves, very little is known about how this photosynthetic adaptation is coordinated with xylem anatomy and vascular architecture.

Another leaf adaptation that allows plants to survive in water-limited niches is elevated bulk hydraulic capacitance at full turgor (*C*_FT_). Bulk *C*_FT_ is defined as the ratio at which Ψ_L_ and relative water content (RWC) decline as leaves dehydrate, multiplied by the total water content per area of leaf (see eqn 1). If leaves from two species experience an identical decline in RWC, the species with higher *C*_FT_ will experience a less severe drop in Ψ_L_. Consequently, leaves with high *C*_FT_ rely less on the influx of water to prevent transpiration-induced drops in Ψ_L_ (Leverett *et al.*, 2023), and are more suited to living in water-limited environmental niches (Smith *et al.*, 1987; Ogburn and Edwards, 2010, 2012; Luo *et al.*, 2021). For example, many succulent species are assumed to have extremely high *C*_FT_, in part from the development of specialized non-photosynthetic water storage tissues which can shrink and expel water to keep the photosynthetic chlorenchyma cells hydrated during drought (Nowak and Martin, 1997; Ahl *et al.*, 2019). As with CAM, very little is known about the vascular adaptations that accompany leaves with elevated *C*_FT_ (Males, 2017; Borland *et al.*, 2018).

Understanding how CAM and *C*_FT_ are coordinated with leaf vascular traits is important for informing attempts to bioengineer the CAM pathway into C_3_ crops. Considerable work is already underway to introduce the CAM cycle into C_3_ species, to make use of the drought tolerance this metabolic pathway confers. In addition to engineering the enzymes needed for the CAM cycle to function (Lim *et al.*, 2019), several auxiliary anatomical adaptations are likely to be required, to ensure that this metabolic pathway can function efficiently. To identify anatomical traits that synergize with CAM, scientists have looked at natural variation to explore ways in which CAM and C_3_ leaves differ. This approach has demonstrated that CAM benefits from large, densely packed succulent mesophyll cells, which are required to store organic acids overnight (Nelson *et al.*, 2005; Barrera-Zambrano *et al.*, 2014; Males 2018). Work is already underway to select C_3_ host plants with succulent mesophyll anatomy, in order to ensure the efficient function of bioengineered CAM pathways (Lim *et al*., 2018, 2020). However, little consideration has been given to the xylem traits that will be needed to optimize the hydraulic physiology of CAM leaves. In addition, changing cell size may increase *C*_FT_, due to transgenic leaves having greater water storage. Therefore, it will also be important to understand whether elevated *C*_FT_ requires changes in vascular architecture. By identifying ways in which CAM and *C*_FT_ are coordinated with xylem anatomy, our findings will aid bioengineering efforts to select/design appropriate host plants with optimal vascular architecture.

To address the relationship between leaf vascular traits, CAM and *C*_FT_, we focused on the genus *Clusia*. These tropical trees, epiphytes and hemiepiphytes exhibit a wide variety of photosynthetic phenotypes, including obligate C_3_ and constitutive CAM species as well as C_3_–CAM intermediates, where CAM accounts for only a fraction of total carbon assimilation (Barrera-Zambrano *et al.*, 2014; Borland *et al.*, 2018; Leverett *et al.*, 2021; Luján *et al.*, 2021, 2023; Pachon *et al.*, 2022). In addition, >5-fold variation in *C*_FT_ exists across *Clusia* (Leverett *et al.*, 2023). CAM and *C*_FT_ appear to be independent traits in this genus, as the former is associated with thick chlorenchyma tissue, whereas the latter is primarily the consequence of investment in adaxial hypodermal hydrenchyma tissue (Leverett *et al.*, 2023). As CAM and *C*_FT_ are independent adaptations in *Clusia* leaves, this genus is ideal for exploring which trait is more influential on vascular architecture.

Xylem conduits within leaves consist of narrow tracheids and wider vessels which, together, conduct water into the distal portions of the lamina. The vasculature of eudicots is typically arranged in a single plane in a leaf, such that larger veins furcate into smaller, finer veins that radiate into the leaf lamina (Esau, 1965; Nelson and Dengler, 1997). The midrib and the two orders of veins that branch from this are known as major veins, and are characterized by thick, structurally reinforced xylem tissue. The finer, branched veins that furcate off the third-order veins (i.e. fourth order and above) are described as minor veins; these typically radiate into the lamina tissue until they eventually end at vein termini (Sack and Scoffoni, 2013). Vein length per area of leaf (VLA), determines a species’ ability to conduct water across the leaf (Boyce *et al.*, 2009; Scoffoni *et al.*, 2011; Sack and Scoffoni, 2013). A higher VLA has two complementary effects: it facilitates the flow of water inside the xylem, whilst also minimizing the distance water needs to travel outside of the xylem (Scoffoni *et al.*, 2017). Consequently, species with higher VLA can more easily conduct water across their leaves (Buckley *et al.*, 2015; Scoffoni *et al*., 2016, 2017). As CAM decreases the rate of water moving into and across leaves, we hypothesized that species with these adaptations would have lower VLA. In addition, elevated *C*_FT_ could have several potential effects on VLA. It is possible that, like CAM, the reduced flux of water through leaves with elevated *C*_FT_ could result in lower VLA. However, it is also possible that species with elevated *C*_FT_ will need greater VLA, in order to increase hydraulic conductance when leaves are rehydrating during brief periods of water availability. Finally, it is possible that *C*_FT_ and VLA are independent: CAM may have a greater impact than elevated *C*_FT_ on the rate of transpiration in *Clusia* (Leverett *et al.*, 2023), which may mean that vascular architecture is optimized more to the former than the latter.

Whilst it is useful to consider vasculature in a two-dimensional (2D) plane, leaves are in fact 3D organs, and the 3D density of veins is determined by both VLA and the thickness of the mesophyll tissue in which they are found (Zwieniecki and Boyce, 2014; Males, 2017). Noblin *et al.* (2008) used both model and real leaves to show that optimal vascular architecture occurs when intervein distance (IVD) roughly equals the vein to lower epidermal distance (IVD ≈ VED). This study predicted that an IVD:VED ratio >1 means insufficient veins were available to efficiently replace the water that was lost from stomata. Conversely, an IVD:VED ratio <1 would mean that superfluous veins are present that do not increase the efficiency of mesophyll hydraulic conductance. Across diverse angiosperm species, IVD and VED were found to be approximately equal, reinforcing the argument that this arrangement is optimal in leaves (Zwieniecki and Boyce, 2014). However, some species with thicker leaves overinvest in veins, meaning they have IVD:VED ratios <1 (de Boer *et al.*, 2016; Males, 2017). Two opposing hypotheses have been proposed to explain this. One suggestion is that greater leaf thickness causes higher hydraulic resistance in the mesophyll apoplast, meaning that leaves need overinvestment in vein placement to maintain efficient hydraulic conductance across the whole leaf (de Boer *et al.*, 2016). A contrasting suggestion is that high *C*_FT_ in thick leaves could require greater vascular conductance in order to quickly refill water reserves following rainfall (Males, 2017). *Clusia* is an ideal model to explore these two alternative hypotheses, as *C*_FT_ is independent of CAM and leaf thickness in this genus. Therefore, it is possible to investigate if high IVD:VED ratios are determined more by CAM/leaf thickness or by *C*_FT_/hydrenchyma thickness.

Based on the aforementioned considerations we conducted interspecific comparisons across the genus *Clusia* to address the following questions:

1. Are CAM and/or elevated *C*_FT_ associated with lower VLA?
2. Are CAM and/or elevated *C*_FT_ associated with IVD:VED ratios <1?

By addressing these questions, we were able to generate a fundamental understanding of the ways in which vascular architecture is coordinated with CAM and *C*_FT_ in leaves.

## MATERIALS AND METHODS

### Species studied

This comparative study investigated a well-characterized and phylogenetically diverse collection of *Clusia* species (Gustafsson *et al.*, 2007; Barrera-Zambrano *et al.*, 2014, and references therein; Leverett *et al*., 2021, 2023). The species studied reflected a diversity of photosynthetic phenotypes: obligate C_3_ species – *Clusia multiflora*, *C. tocuchensis* and *C. grandiflora*; the intermediate/facultative CAM species – *C. lanceolata*, *C. aripoensis*, *C. pratensis* and *C. minor*; and constitutive CAM species – *C. rosea*, *C. fluminensis*, *C. alata* and *C. hilariana*.

### Plant growth conditions

Plants were grown in a glasshouse in Cockle Park farm, as a part of Newcastle University’s *Clusia* collection. The 3–6-year-old plants (~60–100 cm tall) were grown in 3:1 (v/v) compost–sand mixture (John Innes No. 2, Sinclair Horticulture Ltd, Lincoln, UK), in 10-L pots. The glasshouse has fitted photosynthetic LED lights (Attis 5 LED plant growth light, PhytoLux, Worcester, UK) allowing plants to receive a minimum 12 h of light a day. The glasshouse temperatures were 25 °C during the day and 23 °C at night.

### Photosynthetic gas exchange

Gas exchange data used in these comparative analyses were extracted from Barrera-Zambrano *et al.* (2014) along with data on *C. pratensis* and *C. fluminensis* from Leverett *et al.* (2021). Net CO_2_ uptake was recorded over 24 h, using a BINOS infrared gas analyser (Walz, Effeltrich, Germany). The proportion of total diel assimilation occurring during the night in well-watered plants (CAM_ww_) and after 9 d of drought (CAM_d_) was used as a quantitative estimate of CAM.

### Leaf water relations

Estimates of leaf bulk hydraulic capacitance (*C*_FT_) were taken from Leverett *et al.* (2023). Pressure–volume curve data were used to calculate *C*_FT_ with the equation


$$\begin{array}{l} C_{FT}=\;\frac{\delta RWC}{\delta {\;\Psi \;}_{L}\;}\;\times \;WMA\;/\;M_{H_{2}O}, \end{array}$$ (1)

where RWC is relative water content, Ψ_L_ is leaf water potential, WMA is the mass of water per leaf area of fully hydrated leaves and *M*_H2O_ is the molar mass of water. *C*_FT_ was standardized by area, rather than by dry mass, as this is most biologically relevant within the context of evapotranspiration rates of leaves.

### Vein length per leaf area (VLA) and intervein distance (IVD)

**Fig. 1.**
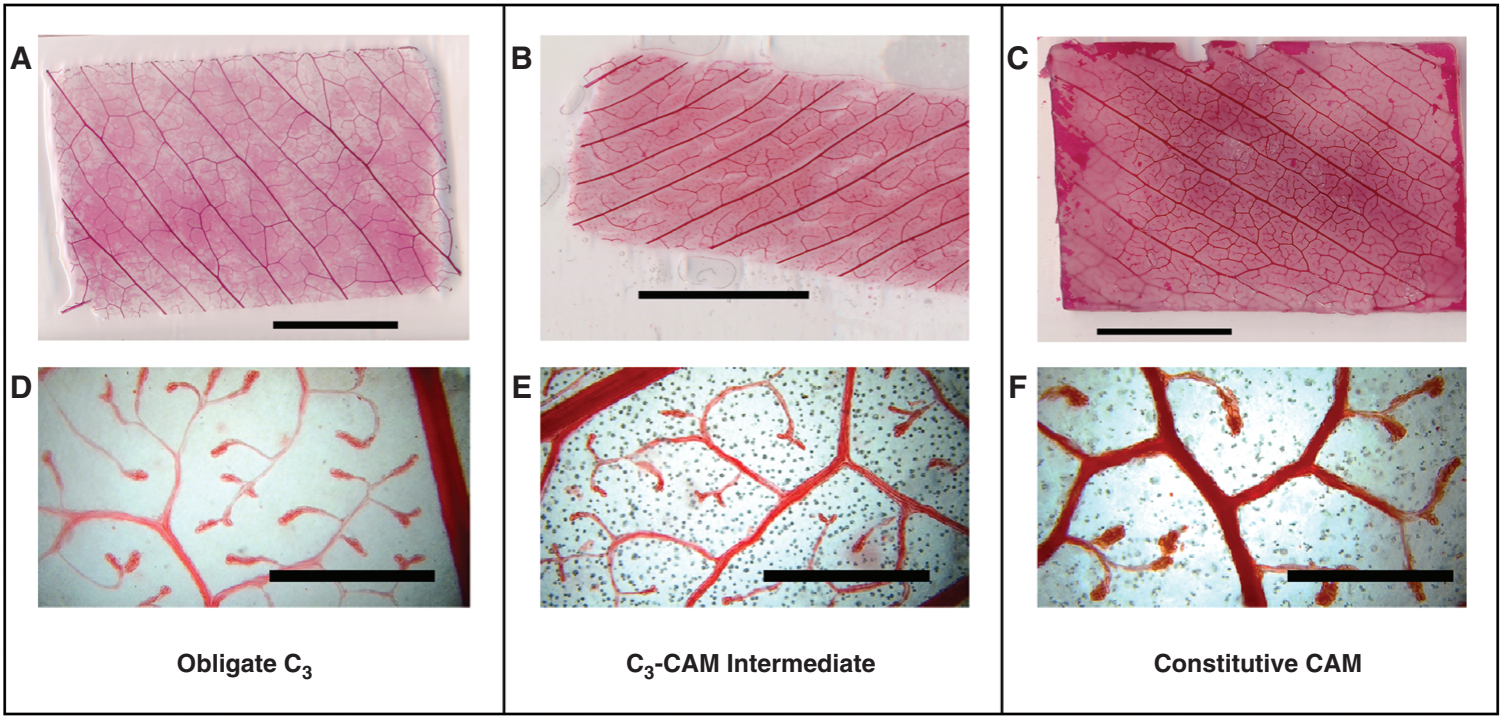
Example images used to estimate major and minor vein length per leaf area (VLA). Example of an obligate C_3_ species (*Clusia multiflora*), a C_3_–CAM intermediate (*C. pratensis*) and a constitutive CAM species (*C. alata*). Scale bars represent 10 mm (A–C), 1 mm (D–F).

Some succulent species are known to have veins organized in multiple planes within a leaf, a phenomenon known as 3D vasculature (Balsamo and Uribe, 1988; Cutler, 2004; Ogburn and Edwards, 2013; Heyduk *et al.*, 2016; Males, 2017; Fradera-Soler *et al.*, 2021; Jolly *et al.*, 2021). Before measuring VLA, leaves were hand-sectioned, to visually check the arrangement of veins. No species of *Clusia* used in this study had 3D vasculature (Supplementary Data Fig. S1). VLA was measured according to the protocol described by Scoffoni *et al.* (2011), with modifications for working with *Clusia* leaves. From each plant, the fourth leaf from the apex was sampled and leaf area was measured using a flatbed scanner (HP Scanjet 5530 Photosmart Scanner, HP, Reading, UK). Primary VLA (i.e. VLA of the midrib) was measured by dividing the length of the midrib by the leaf area. Due to the thick, waxy leaves of *Clusia* it was not possible to clear whole leaves to measure VLA, so instead, for each leaf, a rectangle (2 × 3 cm, or 1 × 2 cm for smaller leaves of *C. lanceolata* and *C. minor*) was cut halfway along the proximal–distal axis of the leaf blade. This rectangle did not include the leaf margin or the midrib. Both abaxial and adaxial surfaces were gently rubbed with a nail file to remove some wax and make fine perforations. Leaf tissue was soaked for 45 min in 3:1 95 % ethanol/acetic acid (Fisher Chemical, Loughborough, UK) to further remove wax. Pigments were then cleared by soaking tissue in 5 % (w/v) NaOH (BDH Chemicals Ltd, Poole, UK) for 45 min. Leaf material was transferred to 50 % (v/v) bleach in aqueous solution for 15 min to remove blackened phenolics. Leaf material was washed in water four times, each for 15 min, and then dehydrated by transfer to solutions containing increasing concentrations of ethanol (dehydration series was 30, 50, 70 and 100 % ethanol, each lasting 20 min). Dehydrated leaf tissue was transferred to a staining solution containing 1 % (w/v) Safranin-O (Sigma Aldrich, St Louis, MO, USA) in 100 % ethanol for 2 min. Leaf tissue was then washed in 100 % ethanol three times, and rehydrated, by repeating the dehydration series in the opposite order. Major veins were imaged using a flatbed scanner (CanoScan 9000F, Cannon LTD, Amsterdam, The Netherlands) at 4800 × 4800-dpi resolution (Fig. 1). Minor veins were imaged using a camera (GXCAM HiChrome-S, GT Vision Ltd, Newmarket, UK) attached to a light microscope (Leitz Diaplan, Stuttgart, Germany). When measuring minor veins, images were acquired at three locations on the leaf tissue, as technical replicates. ImageJ (NIH) was used to measure VLA on images. Total leaf VLA was calculated as the sum of major and minor VLA. Total VLA was used to calculate intervein distance using the equation


$$\begin{array}{l} IVD=\;\frac{1}{VLA\;}. \end{array}$$ (2)

The same leaf used to measure VLA was hand-sectioned to determine the average distance from vein to lower epidermal surface and the thickness of hydrenchyma tissue. Hand-sectioned material was imaged using a camera (Q-IMAGING, QICAM, fast 1394, Surrey, Canada) attached to a fluorescence microscope (Leica DMRB, Wetzlar, Germany) under blue light. As veins are in one plane in *Clusia* leaves (Supplementary Data Fig. S1), VED was calculated as the distance from the middle of a vein to the lower epidermal surface, averaged for three technical replicates per leaf. For each species, seven to nine replicate leaves were used. *Clusia* leaves are hypostomatous, so the distance from veins to upper epidermis was not measured. The presence of a gel-like substance in *C. rosea* leaves prevented acquisition of clear images, so this species was omitted from this analysis (Supplementary Data Fig. S2).

### Statistics

All statistics were performed using R version 4.1.2.

## RESULTS

### Question 1: Are CAM and/or elevated *C*_FT_ associated with lower VLA?

**Fig. 2.**
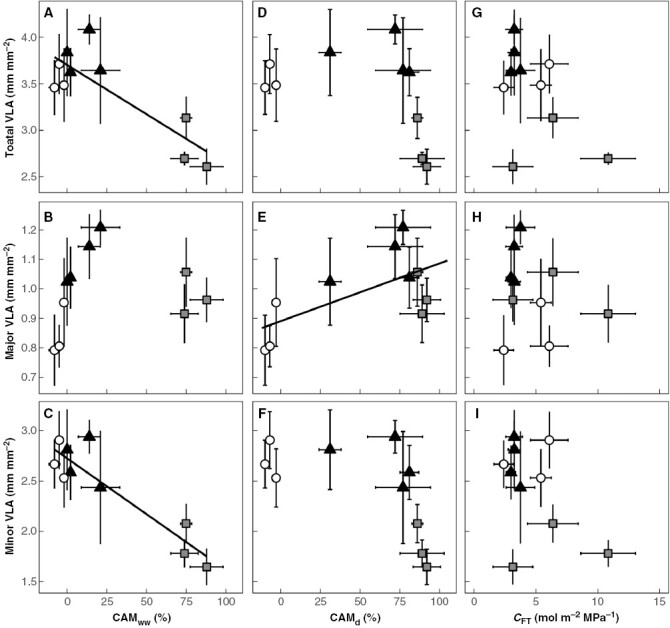
CAM species have lower vein length per leaf area (VLA), due to a lower density of minor veins. (A) A negative correlation exists between the proportion of diel CO_2_ assimilation occurring at night in well-watered plants (CAM_ww_) and total VLA (linear regression: *R*^2^ = 0.66, *P* = 0.003). (B) CAM_ww_ does not correlate with major VLA (linear regression: *P* = 0.69). (C) CAM_ww_ negatively correlates with minor VLA (linear regression: *R*^2^ = 0.80, *P* < 0.001). (D) The percentage of CO_2_ assimilation occurring at night in drought-treated plants (CAM_d_) does not correlate with total VLA (linear regression: *P* = 0.24). (E) CAM_d_ positively correlates with major VLA (linear regression: *R*^2^ = 0.33, *P* = 0.05). (F) CAM_d_ does not correlate with minor VLA (*P* = 0.06). (G) Bulk hydraulic capacitance (*C*_FT_) does not correlate with total VLA (linear regression: *P* = 0.15). (H) *C*_FT_ does not correlate with major VLA (linear regression: *P* = 0.53). (I) *C*_FT_ does not correlate with minor VLA (linear regression: *P* = 0.19). White circles = obligate C_3_ species, black triangles = C_3_–CAM intermediates, grey squares = constitutive CAM species. Error bars are ±1 standard deviation and for each species, *n* = 7–9.

**Fig. 3.**
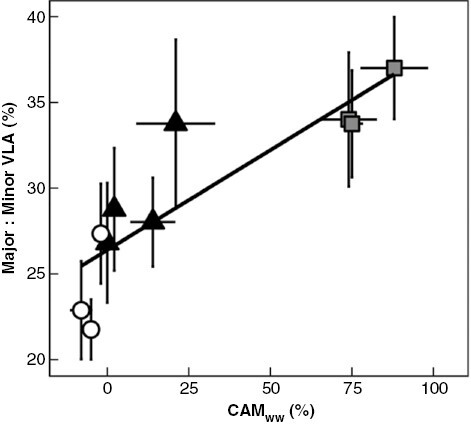
Major veins make up a greater proportion of total VLA in CAM species. The proportion of diel CO_2_ assimilation occurring at night in well-watered plants (CAM_ww_) positively correlates with the percentage of total VLA comprising major veins (percentage major VLA) (linear regression: *R*^2^ = 0.73, *P* = 0.001). White circles = obligate C_3_ species, black triangles = C_3_–CAM intermediates, grey squares = constitutive CAM species. Error bars are ±1 standard deviation and for each species, *n* = 7–9.

We analysed ten species of *Clusia* to determine if CAM and/or elevated *C*_FT_ were associated with lower VLA. Interspecific comparisons found that species that do a greater proportion of their CO_2_ assimilation at night under well-watered conditions (CAM_ww_), had significantly lower total VLA (Fig. 2A). Separate analysis of the major and minor VLA found that only the latter significantly correlated with CAM_ww_ (Fig. 2C). No correlation was found between CAM_ww_ and major VLA (Fig. 2B). As minor VLA, but not major VLA, is lower in species that do CAM, the percentage of total VLA that comprised major veins showed a significant positive correlation with CAM_ww_ (Fig. 3). In contrast, the proportion of CO_2_ assimilation occurring at night under drought-treated conditions (CAM_d_) did not correlate with total VLA (Fig. 2D) or minor VLA (Fig. 2F). However, CAM_d_ positively correlated with major VLA (Fig. 2E). Interspecific variation in *C*_FT_ did not correlate with total, major or minor VLA (Fig. 2G–I). Taken together, these data suggest low VLA is found in species with constitutive CAM phenotypes, but not in species that do facultative CAM, or in species with elevated *C*_FT_. CAM_ww_ and *C*_FT_ are strongly associated with leaf and hydrenchyma thickness, respectively (Barrera-Zambrano *et al.*, 2014; Leverett *et al.*, 2023). Consequently, leaf thickness negatively correlated with total VLA and minor VLA (Supplementary Data Fig. S3). In addition, no correlation was observed between hydrenchyma thickness and total, major or minor VLA (Fig. S4).

**Fig. 4.**
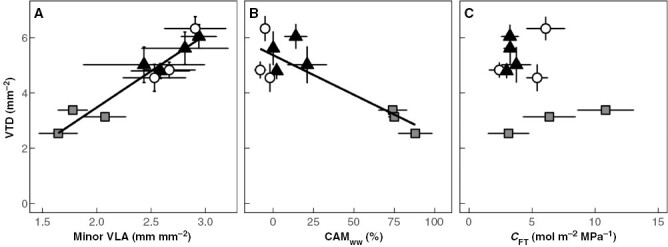
CAM species have lower leaf vein termini density (VTD), due to their lower minor vein length per leaf area (minor VLA). (A) Minor VLA positively correlates with VTD (linear regression: *R*^2^ = 0.90, *P* < 0.001). (B) The proportion of diel CO_2_ assimilation occurring at night (CAM_ww_) negatively correlates with VTD (linear regression: *R*^2^ = 0.71, *P* = 0.001). (C) Bulk hydraulic capacitance (*C*_FT_) does not correlate with VTD (linear regression: *P* = 0.37). White circles = obligate C_3_ species, black triangles = C_3_–CAM intermediates, grey squares = constitutive CAM species. Error bars are ±1 standard deviation and for each species, *n* = 7–9.

Minor veins often end at vein termini (Fig. 1D–F), at which point all water moves out of the xylem and into the mesophyll tissue. We suspected that minor VLA and vein termini density (VTD) would positively correlate with each other. Analysis of ten *Clusia* species confirmed this hypothesis, as minor VLA correlated significantly with VTD (Fig. 4A). In addition, CAM_ww_ negatively correlated with VTD (Fig. 4B). No correlation was found between *C*_FT_ and VTD. Furthermore, leaf thickness negatively correlated with VTD (Supplementary Data Fig. S4), whereas hydrenchyma thickness did not correlate with VTD (Fig. S4).

### Question 2: Are CAM and/or elevated *C*_FT_ associated with IVD:VED ratios <1?

**Fig. 5.**
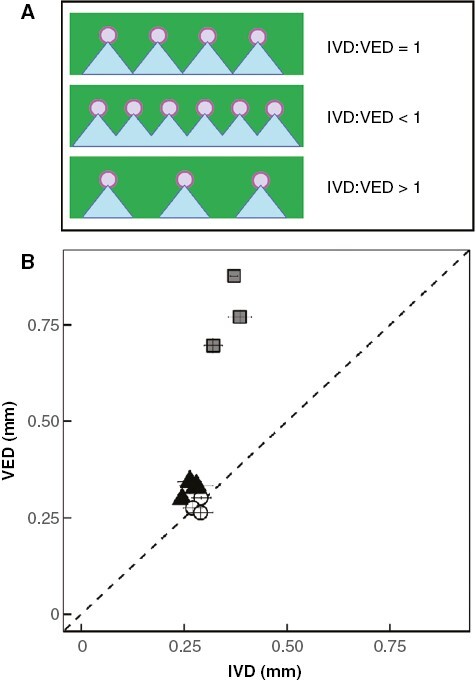
Coordination of intervein distance (IVD) and vein to lower epidermal distance (VED) in leaves. (A) In most angiosperm species, optimal arrangement of veins (purple) in leaves (green) is one where IVD:VED = 1. In this scenario, water (blue) can permeate efficiently into the abaxial mesophyll tissue. When IVD:VED < 1, superfluous veins are present, which do not increase the efficiency with which water permeates into the abaxial mesophyll. When IVD:VED > 1, insufficient veins are present to efficiently replace water lost via abaxial stomata. (B) Across ten species of *Clusia*, species exist where IVD:VED < 1. White circles = obligate C_3_ species, black triangles = C_3_–CAM intermediates, grey squares = constitutive CAM species. Error bars are ±1 standard deviation and for each species, *n* = 7–9.

**Fig. 6.**
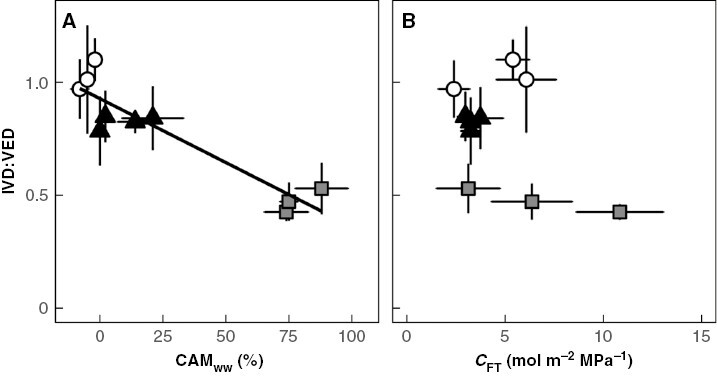
The ratio of intervein distance and vein to epidermis distance (IVD:VED) is higher in CAM species than in C_3_ species. (A) The proportion of diel CO_2_ assimilation occurring at night (CAM_ww_) negatively correlates with IVD:VED ratios across ten species of *Clusia* (linear regression: *R*^2^ = 0.83, *P* < 0.001). (B) Bulk hydraulic capacitance (*C*_FT_) does not correlate with IVD:VED ratios (linear regression: *P* = 0.18). White circles = obligate C_3_ species, black triangles = C_3_–CAM intermediates, grey squares = constitutive CAM species. Error bars are ±1 standard deviation and for each species, *n* = 7–9.

Unlike the majority of angiosperms, some *Clusia* species have IVD:VED ratios <1: a phenomenon termed ‘vascular overinvestment’ (de Boer *et al.*, 2016) (Fig. 5). Across ten species of *Clusia*, IVD:VED ratios positively correlated with CAM_ww_. In contrast, no correlation was observed between *C*_FT_ and IVD:VED ratios (Figs 5 and 6). In addition, IVD:VED ratios positively correlated with leaf thickness, but did not correlate with hydrenchyma thickness (Supplementary Data Fig. S5).

## DISCUSSION

### Leaf vascular architecture is coordinated with constitutive CAM

Both CAM and elevated *C*_FT_ reduce the rate at which water moves through leaves. However, in *Clusia*, VLA appears to have been optimized to match the reduced hydraulic demands conferred by CAM, rather than elevated *C*_FT_. *Clusia* species with constitutive CAM will experience relatively low transpiration rates over the entire year, during both the dry and wet seasons (Holtum *et al.*, 2004; Leverett *et al.*, 2021). By sustaining low transpiration rates, constitutive CAM species require less water to move through the xylem in order to keep leaves hydrated. As constitutive CAM species require lower hydraulic conductance, they develop lower VLA and VTD than C_3_ relatives, meaning less energy needs to be expended on the development of vascular tissue. Low VLAs and VTDs are achieved by developing lower minor VLA (Fig. 2C), as minor veins are less structurally reinforced and more vulnerable to physical damage during drought (Sack and Scoffoni, 2013). In contrast, whilst the C_3_–CAM *Clusia* species can facultatively switch on or upregulate CAM during the dry season, they spend most of the year predominantly doing C_3_ photosynthesis, which will result in higher transpiration rates. Interestingly, C_3_–CAM species did not have intermediate values of total VLA or VTD, but instead their total VLA and VTD values more closely resembled those of obligate C_3_ species (Fig. 2A). It seems that C_3_–CAM species develop higher VLAs and VTDs in order to tolerate the greater rates of transpiration that they are thought to experience most of the year, rather than to optimize vascular architecture for acute dry seasons when they switch to CAM.

In addition to exploring differences in VLA, we estimated the ratio of major/minor VLA across 10 species of *Clusia*. The ratio of major/minor VLA correlated significantly with CAM_ww_, with obligate C_3_ species having the lowest values and constitutive CAM species having the highest (Fig. 3). Unlike total VLA, major/minor VLA ratios appear to occupy intermediate values in C_3_–CAM species. Major and minor veins will behave differently when Ψ_L_ drops, as the latter have been shown to deform, collapsing inwards and restricting water flow (Zhang *et al*., 2016, 2022). Conduit collapse in minor veins has been suggested to act as a hydraulic ‘circuit breaker’, reducing the flow of water and protecting upstream major veins from experiencing higher xylem tensions when leaves dehydrate, for example when vapour pressure deficits (VPD) increase. It is interesting to speculate whether CAM impacts the adaptive benefit of such a circuit-breaker system. The stomata of CAM plants will be open during the night, when it is cooler and more humid, and will be (partially or fully) closed during the driest periods of the day. As a result, CAM plants may be less susceptible to sudden increases in VPD, and therefore their major veins may be less reliant on minor vein collapse for protection. This scenario could explain why major/minor VLA ratios are greater in species with higher values of CAM_ww_. However, it is important to note that CAM species of *Clusia* occur in drier niches, and hence may experience lower Ψ_L_, particularly during the dry seasons (Leverett *et al.*, 2021). This could have the opposite effect, making minor vein collapse more beneficial to C_3_–CAM and constitutive CAM species. More work is needed to understand if CAM is affecting minor vein collapse, and how this varies across the different climatic niches that *Clusia* occupies.

In contrast to CAM, *C*_FT_ did not correlate with either VLA or VTD (Figs 2 and 4). CAM appears to have a greater influence on vascular architecture, despite *Clusia* leaves exhibiting >5-fold variation in *C*_FT_. This observation is somewhat puzzling, because elevated *C*_FT_ would allow transpiring leaves to maintain a more stable Ψ_L_ with less reliance on xylem hydraulic conductivity. Reduced reliance on xylem conductivity could, hypothetically, require lower VLA and VTD. The lack of any correlation between VLA or VTD with *C*_FT_ may be because CAM has a far greater impact on the flux of water through and out of leaves. Inferences from the distribution of *Clusia* suggest that this may be the case: across a precipitation gradient, CAM correlates with increased aridity, whereas hydrenchyma thickness (which confers elevated *C*_FT_ more than other mesophyll tissues) does not (Leverett *et al.*, 2021). This ecological pattern suggests that the water-conservation benefits of CAM are greater than those conferred by elevated *C*_FT_. In addition, ecophysiological modelling has demonstrated that, in *Clusia*, the presence of CAM largely obviates the effect of elevated *C*_FT_ on transpiration rates (Leverett *et al.*, 2023). As a result, interspecific variation in CAM is likely to have a greater impact than elevated *C*_FT_ on the rate at which water traverses leaves. Such a scenario would explain why VLA and VTD are coordinated with CAM and not with *C*_FT_.

In addition to exploring VLA, we also estimated IVD:VED ratios across species of *Clusia* to understand how vein density is coordinated with leaf thickness. In contrast to most angiosperms (Zwieniecki and Boyce, 2014), some species of *Clusia* had IVD:VED ratios <1 (Fig. 4). Similar ‘vascular over-investment’ has been observed in other taxa and appears to be associated with thicker leaves. However, it is unclear if vascular over-investment is an adaptation to overcome the longer apoplastic distance imposed by greater leaf thickness, or if IVD:VED ratios <1 occur in order to facilitate quick refilling of water stores that are responsible for high *C*_FT_ (de Boer *et al.*, 2016; Males, 2017). In *Clusia*, CAM/leaf thickness are independent of *C*_FT_/hydrenchyma thickness, making this genus ideal for exploring these opposing hypotheses (Barrera-Zambrano *et al.*, 2014; Leverett *et al.*, 2023). Our data show that IVD:VED ratios correlate with CAM and leaf thickness (Fig. 6; Supplementary Data Fig. S5), and not with *C*_FT_ or hydrenchyma thickness. In CAM species, thick, succulent photosynthetic chlorenchyma tissue is required to provide adequate space for nocturnal storage of malic acid (Barrera-Zambrano *et al.*, 2014; Males, 2018; Töpfer *et al.*, 2020). However, this will increase the apoplastic distance through which water must travel, in order to infiltrate the entire leaf. As a result, it is likely that CAM species require IVD:VED ratios <1, in order to provide this additional water to the mesophyll and keep the leaf hydrated when stomata are open.

### Learning from nature: xylem traits should be optimized for CAM biodesign

Due to the effects of global warming, aridity will increase across much of the world’s arable land. Therefore, it is paramount that scientists develop novel approaches to increase drought tolerance in crops (Borland *et al.*, 2015; Cushman *et al.*, 2015; Leakey *et al.*, 2019; Pan *et al.*, 2021). To this end, CAM plants have a great deal of potential, as their low transpiration rates and elevated water-use efficiency allow them to tolerate conditions in marginal land that is less amenable to C_3_ or C_4_ crops (Borland *et al.*, 2014). Furthermore, as the majority of crops do not do CAM, considerable efforts are underway to bioengineer this pathway into C_3_ species, to prepare for hotter, drier futures. Beyond introducing the key enzymes that comprise the CAM pathway (Lim *et al.*, 2019), it is essential that the anatomy of host plants is appropriate for CAM to function efficiently. To this end, the introduction of a grape helix–loop–helix transcription factor (*VvCEB1*) has been shown to increase cell size in *Arabidopsis thaliana*, which will provide adequate space for nocturnal accumulation of malate (Lim *et al*., 2018, 2020). However, little attention has been given to the xylem adaptations associated with CAM, and how these vascular traits might be optimized to maximize water use efficiency in bioengineered CAM plants (Borland *et al.*, 2018). The data presented in this study suggest that low VLA and VTD, alongside IVD:VED ratios <1, should be selected when choosing a host plant for CAM bioengineering. It is possible that the *35S::VvCEB1* overexpression lines engineered by Lim *et al.* (2018) exhibit lower VLA and/or higher IVD:VED ratios, due to their wider leaves and larger mesophyll cells. However, xylem anatomy remains unreported for these transgenic plants. If the optimal vascular architecture is not found in *35S::VvCEB1* plants, other manipulations could be incorporated to achieve this goal, such as using leaf-specific promoters to manipulate the auxin signalling pathway (Perico *et al.*, 2022). For example, upregulating cyclophilin 1 *cis*/*trans* isomerase (CYP1), alongside a *35S::VvCEB1*, could be used to remove repression of auxin response factors (ARFs) during leaf development, and generate lower VLAs (Andrade *et al.*, 2022). Future bioengineering efforts must look beyond mesophyll anatomy, to ensure optimal vascular traits are introduced alongside the core enzymes that catalyse the CAM cycle.

## CONCLUSIONS

### Towards a complete anatomical characterization of *Clusia*

The data presented here represent the first characterization of vascular anatomy associated with CAM photosynthesis at the taxonomic level of the genus. The present study demonstrates that CAM is not only associated with the anatomy of photosynthetically active tissues in *Clusia* (Barrera-Zambrano *et al.*, 2014; Luján *et al.*, 2021), but also coordinated with vascular architecture; the low transpiration rates resulting from CAM are associated with low VLAs and VTDs. In addition, succulent photosynthetic tissue in CAM species appears to require high IVD:VED ratios, which we hypothesize is an adaptation to efficiently provide water to the mesophyll in thick leaves. These findings indicate that CAM requires a complex suite of anatomical changes, to optimize both the photosynthetic and hydraulic needs of the leaf.

## SUPPLEMENTARY DATA

Supplementary data are available online at https://academic.oup.com/aob and consist of the following.

Fig. S1: Veins develop in one plane in *Clusia* leaves.

Fig. S2: Gel-like substance prevented clear vein density images from being acquired for *Clusia rosea*.

Fig. S3: Relationships between VLA, leaf thickness and hydrenchyma thickness across ten *Clusia* species.

Fig. S4: Relationship between leaf vein termini density, leaf thickness and hydrenchyma thickness across ten species of *Clusia*.

Fig. S5: Relationship between IVD:VED ratios, leaf thickness and hydrenchyma thickness across ten species of *Clusia*.

mcad035_suppl_Supplementary_Material
