# Supplementary material for: Leaf vein density correlates with crassulacean acid metabolism, but not hydraulic capacitance, in the genus Clusia
Source: Ann Bot. 2023 Feb 23;132(4):801–10. doi: 10.1093/aob/mcad035 (PMC10799986; doi:10.1093/aob/mcad035)
Supplement: mcad035_suppl_Supplementary_Material [file mcad035_suppl_supplementary_material.docx]

**Leaf Vein Density Correlates With Crassulacean Acid Metabolism, But Not Hydraulic Capacitance, In The Genus *Clusia***

**Supplementary Figures**


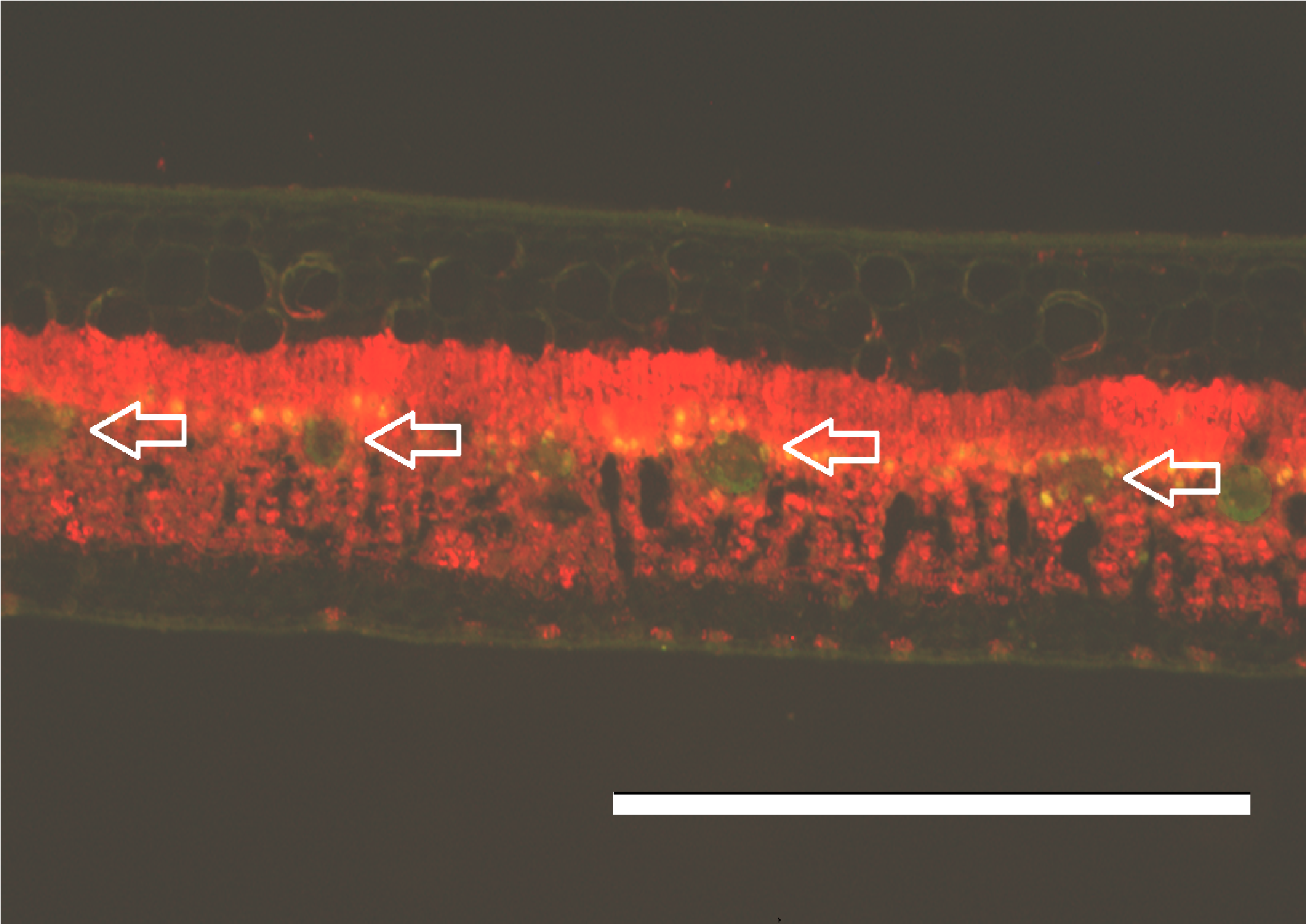


**Fig S1:** Veins develop in one plane in *Clusia* leaves. Image shows the mesophyll of *C. tocuchensis*, with arrows pointing to the veins, which all fall in one flat plane in the leaf. All species in this study had veins in one, flat plane between the palisade and spongy mesophyll tissue layers. Scale bar = 1 mm.


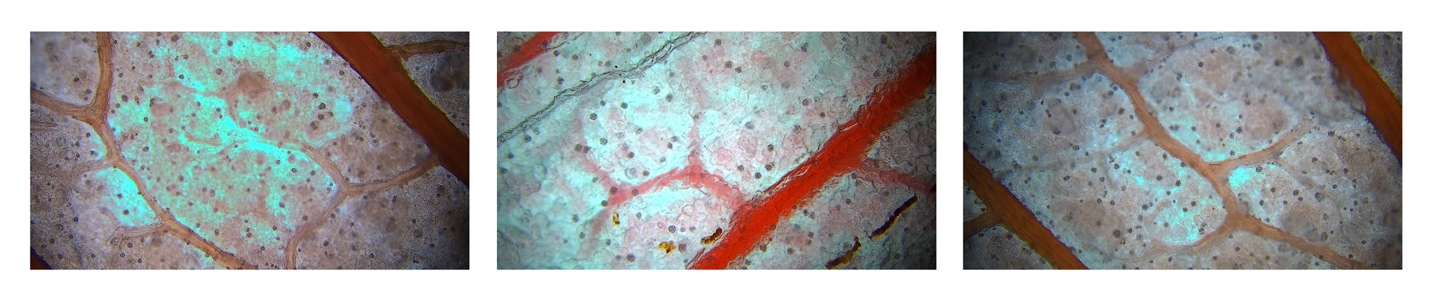


**Fig. S2:** Gel-like substance prevented clear vein density images from being acquired for *Clusia rosea*. Higher order veins were visible but smaller, lower order veins were obscured.


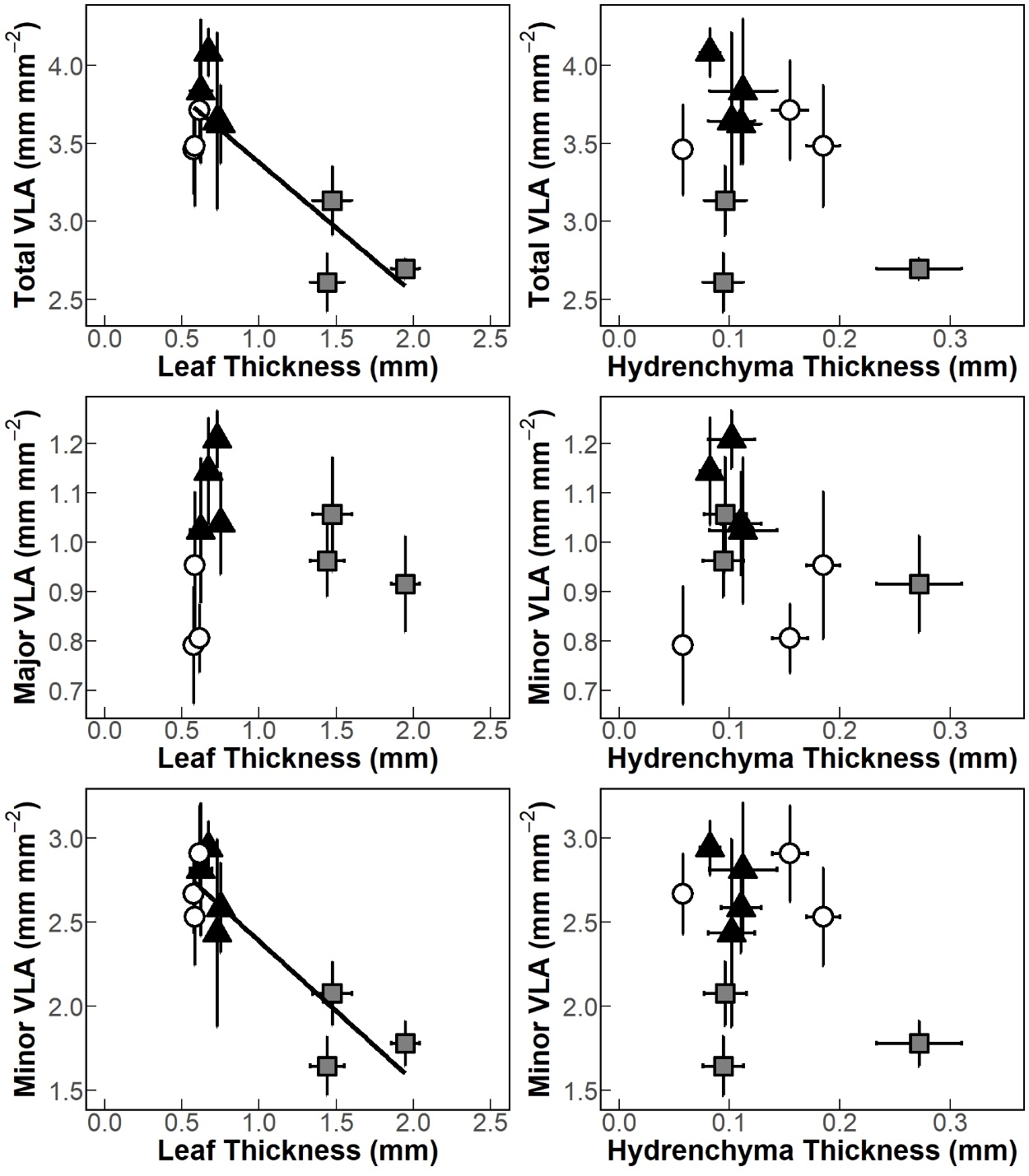


**Fig. S3:** Relationships between VLA, leaf thickness and hydrenchyma thickness across 10 *Clusia* species. **(A)** A negative correlation exists between the leaf thickness and total VLA (linear regression: R^2^ = 0.71, p = 0.001). (**B)** Leaf thickness does not correlate with major VLA (linear regression: p = 0.94). **(C)** Leaf thickness ­negatively correlates with minor VLA (linear regression: R^2^ = 0.77, p < 0.001). **(D)** Hydrenchyma thickness does not correlate with total VLA (linear regression: 0.26). **(E)** Hydrenchyma thickness does not correlate with major VLA (linear regression: 0.48). **(F)** Hydrenchyma thickness does not correlate with minor VLA (linear regression: 0.35). White circles = obligate C_3_ species, black triangles = C_3_-CAM intermediates, grey squares = constitutive CAM species. Error bars are ± 1 standard deviation and for each species, n = 7-9


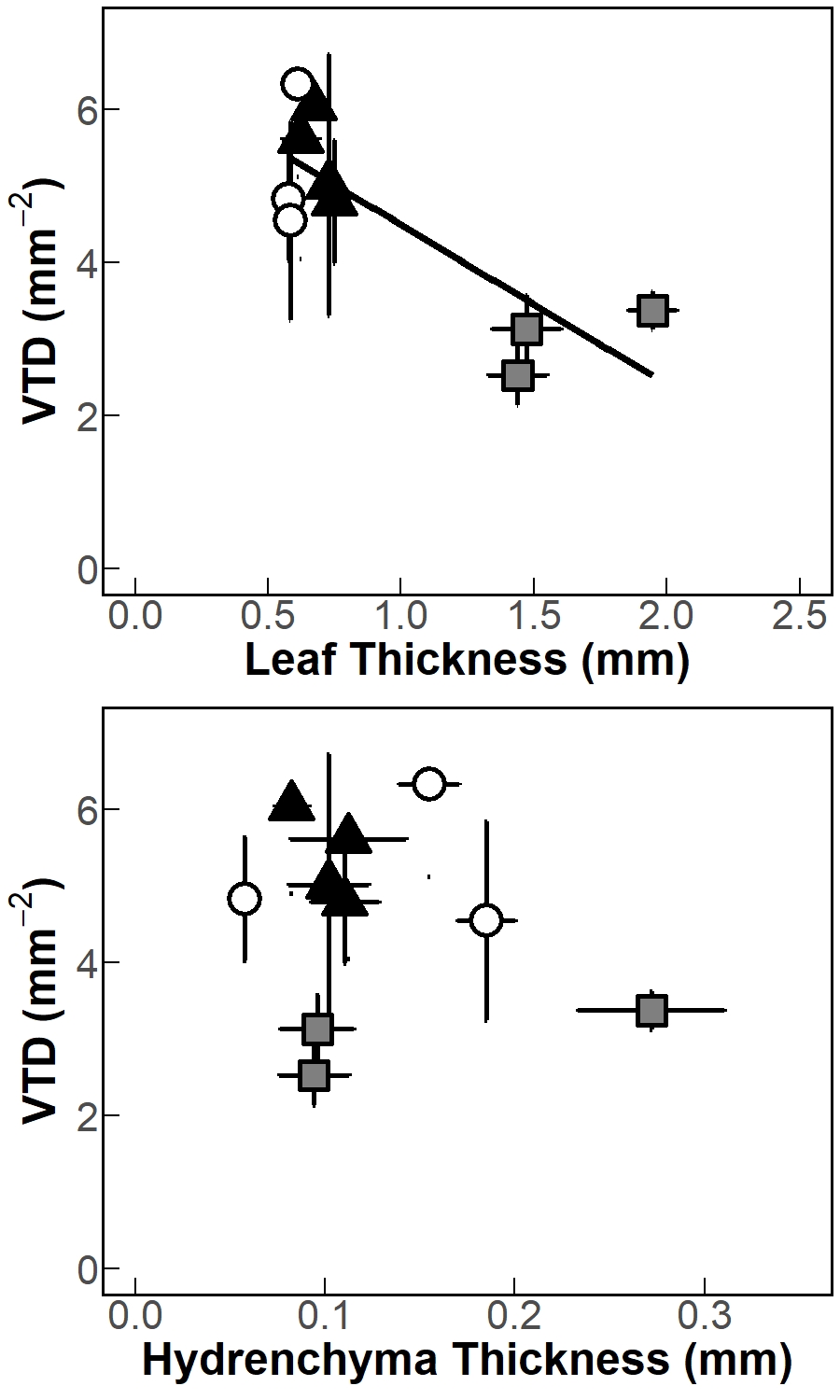


**Fig. S4:** Relationship between leaf vein termini density (VTD), leaf thickness and hydrenchyma thickness across 10 species of *Clusia*. **(A)** Leaf thickness negatively correlates with VTD (linear regression: R^2^ = 0.61, p = 0.004) **(B)** Hydrenchyma thickness does not correlate with VTD (linear regression: p = 0.62). White circles = obligate C_3_ species, black triangles = C_3_-CAM intermediates, grey squares = constitutive CAM species. Error bars are ± 1 standard deviation and for each species, n = 7-9.


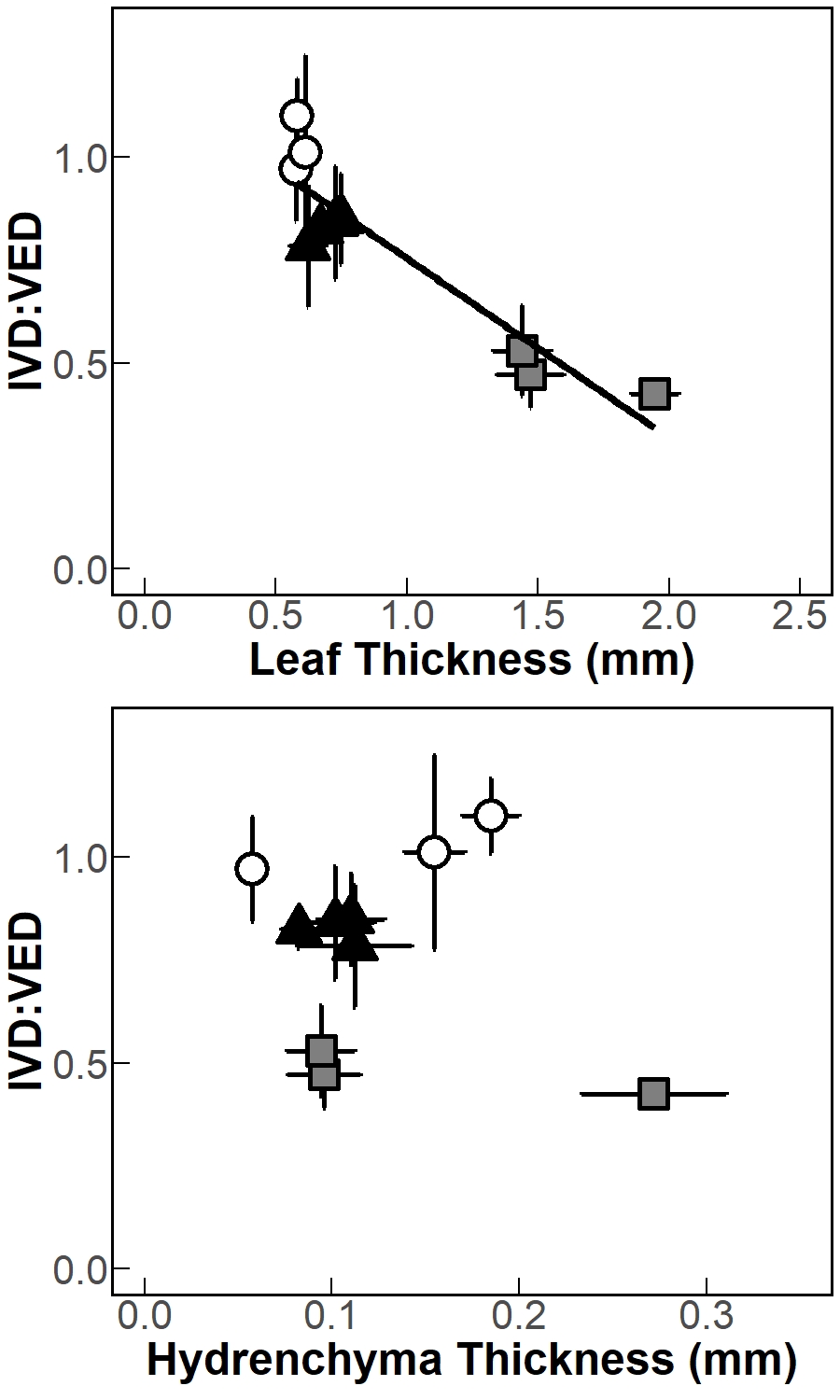


**Fig. S5:** Relationship between IVD:VED ratios, leaf thickness and hydrenchyma thickness across 10 species of *Clusia*. **(A)** Leaf thickness negatively correlates with IVD:VED ratio (linear regression: R^2^ = 0.83, p < 0.001). **(B)** Hydrenchyma thickness does not correlate with IVD:VED ratio (linear regression: p = 0.57). White circles = obligate C_3_ species, black triangles = C_3_-CAM intermediates, grey squares = constitutive CAM species. Error bars are ± 1 standard deviation and for each species, n = 7-9.
